# Screen-detected *vs* clinical breast cancer: the advantage in the relative risk of lymph node metastases decreases with increasing tumour size

**DOI:** 10.1038/sj.bjc.6602289

**Published:** 2004-12-14

**Authors:** L Bucchi, A Barchielli, A Ravaioli, M Federico, V De Lisi, S Ferretti, E Paci, M Vettorazzi, S Patriarca, A Frigerio, E Buiatti, and the SCREENREG Working Group

**Affiliations:** 1 Romagna Cancer Registry, Luigi Pierantoni Hospital, Forlì 47100, Italy; 2 Epidemiology Unit, ASL 10, Firenze, Italy; 3 Modena Cancer Registry, Modena, Italy; 4 Parma Cancer Registry, Parma, Italy; 5 Ferrara Cancer Registry, Ferrara, Italy; 6 Tuscany Cancer Registry/Epidemiology Unit, CSPO, Firenze, Italy; 7 Veneto Cancer Registry, Padova, Italy; 8 Piedmont Cancer Registry, CPO Piemonte, Torino, Italy; 9 First Screening Centre, Torino, Italy; 10 Tuscany Regional Health Agency, Firenze, Italy

**Keywords:** breast carcinoma, screening, mammography, lymph nodes, natural history

## Abstract

Screen-detected (SD) breast cancers are smaller and biologically more indolent than clinically presenting cancers. An often debated question is: if left undiagnosed during their preclinical phase, would they become more aggressive or would they only increase in size? This study considered a registry-based series (1988–1999) of 3329 unifocal, pT1a-pT3 breast cancer cases aged 50–70 years, of which 994 were SD cases and 2335 clinical cases. The rationale was that (1) the average risk of lymph node involvement (N+) is lower for SD cases, (2) nodal status is the product of biological aggressiveness and chronological age of the disease, (3) for any breast cancer, tumour size is an indicator of chronological age, and (4) for SD cases, tumour size is specifically an indicator of the duration of the preclinical phase, that is, an inverse indicator of lead time. The hypothesis was that the relative protection of SD cases from the risk of N+ and, thus, their relative biological indolence decrease with increasing tumour size. The odds ratio (OR) estimate of the risk of N+ was obtained from a multiple logistic regression model that included terms for detection modality, tumour size category, patient age, histological type, and number of lymph nodes recovered. A term for the detection modality-by-tumour size category interaction was entered, and the OR for the main effect of detection by screening *vs* clinical diagnosis was calculated. This increased linearly from 0.05 (95% confidence interval: 0.01–0.39) in the 2–7 mm size category to 0.95 (0.64–1.40) in the 18–22 mm category. This trend is compatible with the view that biological aggressiveness of breast cancer increases during the preclinical phase.

The objectives of mammography screening are to detect small tumours and prevent them from growing to a larger size and becoming lethal (Tabar *et al*, 1999). As effectiveness of early detection in reducing breast cancer mortality has been demonstrated, there seems to be no doubt that this is the case for a significant proportion of screen-detected (SD) cases. However, SD cancers are not only smaller than clinically presenting cancers but also more indolent biologically as they are lower histological grade, express p53 and Ki-67 nuclear proteins less frequently, and have fewer mitotic cells, more moderate/rich oestrogen and progesterone receptor levels, and lower levels of microvessel density (Uyterlinde *et al*, 1991; Hakama *et al*, 1995; Moezzi *et al*, 1996; Tabar *et al*, 1999; Groenendijk *et al*, 2000; Ernst *et al*, 2002). An often debated question (Thomas, 1995) is: if left undiagnosed during their preclinical phase, would they become biologically more aggressive or would they only increase in size?

The only objective method to determine whether biological behaviour of preclinical breast cancer worsens over time is to periodically take and analyse cell samples or tissue specimens from SD lesions surgically untreatable. As no such observations have ever been reported, current knowledge is based on cross-sectional comparisons of SD lesions with clinically presenting tumours.

The SCREENREG study was conducted by a group of Italian cancer registries to evaluate the effect of mammography screening on the trends in stage-specific incidence of breast cancer. Among its secondary objectives was to investigate the question of biological progression of the preclinical disease.

## MATERIALS AND METHODS

General methods of the SCREENREG study are reported in detail elsewhere (Buiatti *et al*, 2002, 2003). In brief, each participating registry contributed a consecutive series of breast cancer cases (International Classification of Diseases for Oncology (ICD-O) topography code 174) registered before and after implementation of the local screening programme. Staging and treatment information was retrospectively retrieved and prospectively collected by trained personnel with a review of original pathology and clinical case records. Data were submitted to the coordinating centre according to a common set of variables. These included the following: original index number; date of birth; date of registration; histological type (ICD-O morphology code); simultaneous bilaterality (yes, no); multifocality (yes, no); surgical treatment (unknown, unperformed, conservative, radical); tumour size (invasive component) in mm; pT category according to tumour, node, metastasis (TNM) classification; number of axillary lymph nodes recovered; number of positive axillary lymph nodes; pN category; distant metastases (yes, no/unknown); and detection modality (SD, death certificate only, clinical diagnosis). Women with simultaneous bilateral cancers were classified according to the lesion with the highest pN (or pT in the case of equal pN).

### Rationale

Starting from the universal observation that the average risk of lymph node involvement is lower for SD breast cancer cases compared with clinically diagnosed cases, the rationale of the current study was based on the following assumptions: (1) axillary lymph node status is the product of biological aggressiveness and chronological age of the disease (Mittra and MacRae, 1991), (2) for any breast cancer case, tumour size is an indicator of its chronological age, and (3) for SD cases, tumour size is specifically an indicator of the duration of the preclinical phase, that is, an inverse indicator of lead time (Anderson *et al*, 1991; Norden *et al*, 1997; Ernst *et al*, 2002). We evaluated the tumour size-specific risk of lymph node involvement for SD cases relative to that for clinical cases. The study hypothesis was that the relative protection of SD cases from the risk of nodal involvement (and thus, their relative biological indolence) decreases with increasing tumour size, that is, with increasing duration of the preclinical phase or decreasing lead time. If this hypothesis was true, then it would be suggested that biological characteristics of breast cancer worsen progressively during the preclinical phase.

As reported elsewhere (Buiatti *et al*, 2003), the SCREENREG study showed an incidence increase of early-stage breast cancer following introduction of screening that was only partially explained by the proportion of SD cases. As this was compatible with a concomitant diffusion of spontaneous screening outside organised programmes, the series of breast cancers registered in the last 5 years prior to screening implementation was considered a more reliable comparison group.

### Case series

The SCREENREG database included 20 258 cases. Selection of eligible cases was based on the following criteria. First, we excluded all records from three cancer registries supplying only prescreening cases or cases with unknown tumour size in mm. The study was restricted to seven provinces situated in northern Italy (Torino, Parma, Modena, Ferrara, Ravenna, Forlì-Cesena, and Rimini). The total female population was about 2 300 000, that is, 8% of Italian women according to 1991 census. The year of registration varied between 1988 and 1999. The year of first implementation of screening for women aged 50–69 years was 1992 for Torino, 1995 for Modena, 1996 for Ravenna and Forlì-Cesena, 1997 for Ferrara and Rimini, and 1998 for Parma. The years of screening covered by the study varied between 1 and 4.

Second, we excluded the clinical cases registered in the years when each local screening programme was ongoing, the cases registered >5 years before the implementation of each programme, and the cases aged <50 or >70 years (a considerable proportion of cases detected by mammography at age 69 years were surgically treated and registered at age 70 years).

Third, we excluded the cases with the following characteristics: registration with death certificate only; ICD-O morphology code of sarcoma, lymphoma, and leukaemia; ICD-O behaviour code 2 or pTis according to TNM classification; tumour size of 1 mm or pT1mic; no evidence of primary tumour or pT0; pT4; presurgical chemotherapy; surgical treatment unperformed or unknown; and multifocality.

There remained 4055 potentially eligible cases of invasive, surgically treated, unifocal, pT1a-pT3 breast carcinoma aged 50–70 years. These comprised 1111 SD cases and 2944 clinically presenting cases. The current analysis considered only those cases undergoing axillary dissection and classified for tumour size in mm, number of lymph nodes recovered, and pN. These numbered 994 (89%) among SD cases and 2335 (79%) among clinical cases, for a total of 3329 (82%).

### Statistical analysis

**Figure 1:**
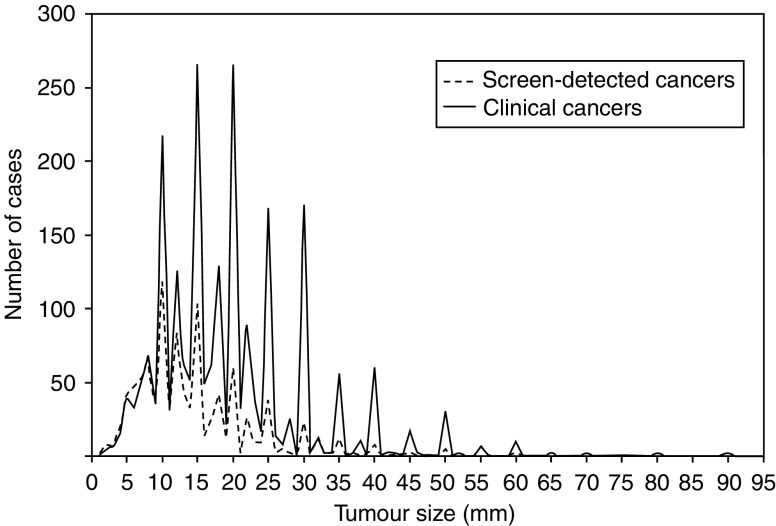
Frequency distribution of study cases (*n*=3329) by detection modality and tumour size.

General characteristics of SD cases were compared with those of clinically diagnosed cases using the Kruskal–Wallis test (distribution by age and number of lymph nodes recovered) and the *χ*^2^ test (distribution by histological type and tumour size). As shown in Figure 1, the frequency distribution of tumour size was found to be compatible with a major phenomenon of rounding-up to the nearest multiple of 5 mm. To reduce biases, tumour size was categorised as 2–7, 8–12, 13–17, 18–22, 23–27, and ⩾28 mm.

As a first step in data analysis, the odds ratio (OR) estimate (with 95% confidence interval (CI)) of the risk of nodal involvement for SD cases compared with clinical cases was calculated for each tumour size category. Total OR was adjusted for tumour size category using the Mantel–Haenszel method. In both groups of cases, the association of tumour size with nodal status was evaluated with the *χ*^2^ test for trend.

A multiple logistic regression model (model #1) was then built that included terms for detection modality, tumour size category, patient age (as a continuous variable), histological type (ductal, lobular, tubular, other), number of lymph nodes recovered (continuous), and registry. These were removed from the model if the likelihood ratio statistic based on the maximum-likelihood estimates had a probability greater than 0.10.

A term for the detection modality-by-tumour size category interaction was then entered. The objective of this second (#2) model was to determine whether the relative risk of lymph node involvement for SD cases compared with clinically detected cases varied in relation to tumour size category. The OR associated with detection modality was calculated as 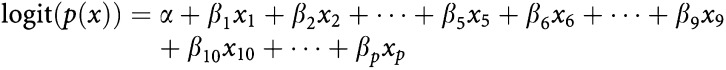 where *x*_1_ is the detection modality, *x*_2_, …, *x*_5_ are the dummy variables for tumour size category, *x*_6_, …, *x*_9_ are the dummy variables for the detection modality-by-tumour size interaction, and *x*_10_ to *x*_*p*_ are the other covariates. Significance of interaction was tested with the deviance *χ*^2^ test.

Adequacy of model #2 was examined with the goodness-of-fit test. In addition, points or cases for which the model did not fit sufficiently were identified with the calculation of the standardised residuals or outliers, the leverage points, and the delta–beta points. As original records could not be retrieved and checked for coding and data-entry errors, all of these were removed (model #3) and analysis was repeated.

Finally, the OR for the main effect of detection by screening in model #3 was computed as exp^(*β*_1_)^ for the 2–7 mm size category, and exp^(*β*_1_+*β_j_*)^ (where *j*=2, …, 5) for the subsequent categories (Kleinbaum and Klein, 2002).

## RESULTS

The median age was 61 years in both groups of cases. SD cancers had a more favourable distribution by tumour size (*P*=0.000), with 73% cases ⩽17 mm *vs* 48%, a greater median number of axillary lymph nodes recovered (17 *vs* 15, *P*=0.000), and a distribution by histological type (ductal, 80%; lobular, 14%; tubular, 3%; other, 2%) similar to that of clinical cases (ductal, 80%; lobular, 13%; tubular, 2%; other, 4%).

Table 1 shows that the average proportion of SD cases with positive lymph nodes, 23%, was lower than that of clinically detected cases, 40%. The crude OR was 0.44. After adjustment for tumour size category, overall protection from the risk of lymph node involvement decreased to 0.62 (95% CI: 0.52–0.75). In both groups, the proportion of node-positive cases was positively associated with tumour size (*P*=0.000). However, it increased more steeply among SD cases. This led the size-specific OR to roughly increase until a diameter of 22 mm.

Table 2 shows the results of the first two logistic models fitted. In model #1, after simultaneous adjustment for tumour size, patient age, number of axillary lymph nodes recovered, and histological type, SD cases showed a significantly lower risk of lymph node involvement (OR=0.59).

Model #2 revealed a significant detection modality-by-tumour size interaction (deviance *χ*^2^=13.78, df=5, *P*=0.017). Although irregularly, the risk of lymph node involvement for SD cases relative to that of clinical cases increased with increasing tumour size. The OR for the effect of detection by screening was 0.33 when the tumour size was 2–7 mm (reference category). Data in the table indicate that the OR was 0.33 × 1.57 (or 0.52) in the 8–12 mm size category, 0.33 × 1.28 in the 13–17 mm size category, and so on.

In model #2, 49 standardised residuals (four of which were also delta–beta points) and 35 leverage points (three of which were also delta–beta points) were identified. After exclusion of these cases, analysis was repeated (model #3) (Table 3). The results showed a stronger detection modality-by-tumour size interaction. With a size of 2–7 mm, detection by screening appeared to be associated with an OR as low as 0.05. The other ORs in the table must be interpreted like those resulting from model #2.

Table 4 gives the final outcome of analysis. The OR for the main effect of detection by screening was computed from the results of model #3. It appears that, taking the detection modality-by-tumour size interaction into account after exclusion of residuals and leverage points, the risk of nodal metastases increased linearly with increasing tumour size and approached unity among cases 18–22 mm in diameter. Although with borderline significance, the OR was below unity for the last two size categories.

## DISCUSSION

As expected, the average proportion of patients with lymph node metastases was lower among SD cases than it was among clinically presenting cases. Moreover, it was positively associated with tumour size in both groups. With increasing tumour size, however, it increased more steeply among SD cases. Accordingly, analysis of interaction demonstrated that the relative risk of lymph node involvement for preclinical cancers increased with increasing tumour size. In other words, their relative advantage was progressively eroded. If our assumption of tumour size of SD cases as a proxy inverse indicator of lead time is valid, then our results are compatible with the interpretation that the biological aggressiveness of breast cancer increases progressively during the preclinical phase.

This does not explain why, after progressively approaching unity, the relative risk of nodal metastases decreased again – although at a borderline level of significance – for SD cases of larger size. Owing to the paucity of such cases (Table 1), their behaviour might be subject to random variation. A similar observation, however, was also reported from the Edinburgh Randomised Breast Screening Project (Anderson *et al*, 1991). The hypothesis we raise points to the fact that most years of screening covered by this study were the initial years of each local programme. As large tumour size suggests short or virtual lead time, the relative risk of nodal metastases for those cases is likely due to the presence of poorly aggressive diseases detected at prevalence screen. If so, a consistent and comprehensive interpretation of results is that (1) a small subset of SD cancers with a relatively stable biological indolence actually exist, (2) they become apparent only among the few large-sized cases detected at first screen but with no significant lead time, and (3) among small, true preclinical SD lesions, their stable biological behaviour is overwhelmed by those cases for which the relative risk of nodal metastases is inversely related to lead time.

Our findings are at variance with some previous studies. In particular, the common statement (Klemi *et al*, 1992; Norden *et al*, 1997; Molino *et al*, 2000; Heimann *et al*, 2002) that the risk of nodal involvement adjusted for tumour size is lower for SD cancers was demonstrated to be misleading, although formally correct. With a size-specific pattern of relative risk such as that shown in Table 1, tumour size qualifies as an interaction factor rather than a confounder. This is equivalent to saying that adjustment for tumour size obscures the real effect of this variable on the relative risk of nodal involvement. Using a study design similar to our own, Tabar *et al* (1987) failed to demonstrate a significant detection modality-by-tumour size interaction. The statistical power of the study, however, was insufficient for this effect to be formally evaluated.

The current investigation confirms one observation reported by Anderson *et al* (1991). In a case series from the Edinburgh Randomised Breast Screening Project, SD cancers had a crude (univariate) advantage in the frequency of positive lymph nodes that decreased progressively from pT1a to pT1c lesions. Moreover, our findings are compatible with those by Ernst *et al* (2002), who observed univariate differences between some biological characteristics of SD and clinical cases that were restricted to lesions ⩽20 mm in diameter. Assuming that lymph node status for any given tumour size reflects the biological aggressiveness of the disease, this study also adds support to the view that malignancy grade of preclinical breast cancer increases with increasing tumour size (Duffy *et al*, 1991; Tabar *et al*, 1999).

Could there be alternative explanations for our results? In the first place, we have to consider that the proportion of potentially eligible cases included into analysis was smaller for clinical cases. The difference, however, was limited (79 *vs* 89%). Moreover, there is no specific reason to believe that the observed trend in the relative risk of nodal involvement reflected a selection bias, if any.

Another problem is that screening may be responsible for overdiagnosis of nonaggressive cancers (Holmberg *et al*, 1992). In a pooled estimate, these accounted for 10–20% of cases detected in three screening trials (Wald *et al*, 1994). In many studies, however, no evidence for overdiagnosis was obtained (Peeters *et al*, 1989; Olsen *et al*, 2003). Most importantly, no published data support an inverse association between overdiagnosis and tumour size, that is, the *conditio sine qua non* for this phenomenon to be considered a potential explanation for our results.

Overdiagnosis may also result from histological misinterpretation of benign lesions as malignant. As small tumours are expected to be highly differentiated, the risk of this type of overdiagnosis occurring is inversely related to lesion size and, thus, is greater among SD cases (Holmberg *et al*, 1992). It clearly appears, however, that size stratification of analysis made this potential problem to have no influence, unless one speculates that impalpability itself conveys a greater risk of misinterpretation. Moreover, the hypothesis that lesions detected by mammography were more likely to be misinterpreted implies that histology evaluation was more accurate in the prescreening years and/or less accurate in the hospitals involved in screening. In fact, an opposite time trend and a higher standard of quality in breast surgery reference hospitals are more conceivable, if any.

One related hypothesis is that study results were biased by differences in accuracy of histopathological staging. The observed relative risk of nodal involvement for small tumours detected by screening might be accounted for by a systematic overestimate of their diameter and/or an opposite mismeasurement of clinically diagnosed lesions. Frequency distribution in Figure 1, however, suggested that major error in tumour size measurement was a random one. We also considered that the risk of nodal involvement in the earliest cases could be biased by differences in detection of microinvasive carcinomas. In fact, we excluded such lesions from both study groups. As to lymph node status, SD cases had a slightly greater median number of lymph nodes recovered (17 *vs* 15). This variable, however, was entered into the multivariate models.

Finally, our results raise questions about mammography sensitivity. It is generally agreed that mammography is more sensitive for indolent lesions (Thomas, 1995). If one assumes that biological characteristics of breast cancer are stable, then our results may be compatible with the explanation that the tendency for mammography to be more sensitive for indolent lesions is concentrated among small tumours and decreases with increasing size. To our knowledge, however, such a hypothesis has never been raised.

Some methodological limitations in the study design need to be pointed out. First, the large size of this multicentre study allowed for a formal analysis of the effect of tumour size on the relative risk of lymph node involvement. However, the case series was not large enough to enable the nodal status to be defined as the number of positive lymph nodes. In particular, the number of SD cases with ⩾4 positive nodes was negligible. In the reference 2–7 mm size category, there were only two such cases.

Second, a linear increase in the relative risk of nodal metastases for SD cases – as one would expect from a ‘natural’ phenomenon – was observed only after removal of residuals, leverage points, and delta–beta points. Unfortunately, we were unable to check the original records for coding and data-entry errors, if any. Exclusion of those cases (model #3) led to an OR as low as 0.05 for the smallest lesions. Although more reliable for our purposes, this estimate must be considered with caution.

Third, we could not compare the two study groups of cases for the biological indicators most commonly used in the literature. The only item of biological information virtually collected by Italian cancer registries, that is, tumour grade, was excluded from the SCREENREG database because of incomplete availability and poor standardization. However, as pointed out in other studies similar to our own (Norden *et al*, 1997; Heimann *et al*, 2002), nodal status is the strongest and most objective single indicator of biological virulence of breast cancer for any given tumour size.

In this study, we attempted to explore one of the most interesting and uncertain theoretical issues of mammography screening. We conclude that our results add further support to the view that biological aggressiveness of breast cancer increases during the preclinical phase. Our observations suggest that if a preclinical breast cancer is left undiagnosed, its biological behaviour worsens before the disease surfaces clinically.

## Figures and Tables

**Table 1:** Univariate analysis of the risk of axillary lymph node metastases

		**Lymph node metastases**				
**Tumour size and detection modality**	**Total no. of cases**	**No**	**Yes**	**(%)**	**Crude OR**	**95% CI**
*2–7 mm*						
Clinical	147	126	21	(14.3)	1.00	Referent
SD	177	167	10	(5.6)	0.36	0.16–0.80
						
*8–12 mm*						
Clinical	479	371	108	(22.6)	1.00	Referent
SD	328	283	45	(13.7)	0.55	0.37–0.80
						
*13–17 mm*						
Clinical	492	301	191	(38.8)	1.00	Referent
SD	219	170	49	(22.4)	0.45	0.31–0.66
						
*18–22 mm*						
Clinical	537	312	225	(41.9)	1.00	Referent
SD	142	81	61	(43.0)	1.04	0.72–1.52
						
*23–27 mm*						
Clinical	249	125	124	(49.8)	1.00	Referent
SD	64	38	26	(40.6)	0.69	0.39–1.21
						
⩾28 mm						
Clinical	431	166	265	(61.5)	1.00	Referent
SD	64	30	34	(53.1)	0.71	0.42–1.21
						
*Total*						
Clinical	2335	1401	934	(40.0)	1.00	Referent
SD	994	769	225	(22.6)	0.44	0.37–0.52

OR=odds ratio; SD=screen detected; CI=confidence interval.

**Table 2:** Multivariate analysis of the risk of axillary lymph node metastases

**Model**	**Covariates of interest^a^**	**P-value**	**Adjusted OR^b^**	**95% CI**
#1^c^	*Detection modality*			
	Clinical		1.00	Referent
	SD	0.000	0.59	0.49–0.71
				
#2^d^	*Detection modality*			
	2–7 mm			
	Clinical		1.00	Referent
	SD	0.006	0.33	0.15–0.73
	*Detection modality-by-tumour size interaction*	0.018		
	8–12 mm			
	SD	0.315	1.57	0.65–3.77
	13–17 mm			
	SD	0.578	1.28	0.54–3.06
	18–22 mm			
	SD	0.013	3.04	1.27–7.29
	23–27 mm			
	SD	0.170	1.97	0.75–5.19
	⩾28 mm			
	SD	0.134	2.07	0.80–5.37

SD=screen detected; CI=confidence interval.

a Both models included also terms for patient age (continuous variable), histological type (ductal, lobular, tubular, other), number of lymph nodes recovered (continuous), and tumour size. The variable ‘registry’ was removed from model #1 as nonsignificantly contributing to its likelihood (*P*=0.6542).

b OR=odds ratio; ORs from model #2 are to be interpreted as follows: when tumour size was 2–7 mm (reference category), the relative risk of lymph node involvement for SD cases was 0.33; when tumour size was 8–12 mm, the relative risk was 0.33 × 1.57, and so on.

c Goodness-of-fit test: Pearson's *χ*^2^=2611.20, df=2631, *P*=0.6043.

d Goodness-of-fit test: Pearson's *χ*^2^=2615.04, df=2626, *P*=0.5565.

**Table 3:** Multivariate analysis of the risk of axillary lymph node metastases after removal of standardised residuals, leverage points, and delta–beta points from model #2 in Table 2

**Model^a^**	**Covariates of interest^b^**	**P-value**	**Adjusted OR^c^**	**95% CI**
#3	*Detection modality*			
	2–7 mm			
	Clinical		1.00	Referent
	SD	0.004	0.05	0.01–0.39
	*Detection modality-by-tumour size interaction*	0.000		
	8–12 mm			
	SD	0.068	7.04	0.87–57.1
	13–17 mm			
	SD	0.051	7.92	0.99–63.2
	18–22 mm			
	SD	0.006	18.70	2.33–149.8
	23–27 mm			
	SD	0.024	11.56	1.38–96.58
	⩾28 mm			
	SD	0.022	12.02	1.44–100.2

SD=screen detected; CI=confidence interval.

a Goodness-of-fit test: Pearson's *χ*^2^=2381.51, df=2497, *P*=0.9506.

b The model included also terms for patient age (continuous variable), histological type (ductal, lobular, tubular, other), number of lymph nodes recovered (continuous), and tumour size.

c OR=odds ratio; ORs are to be interpreted as those from model #2 (Table 2).

**Table 4:** Risk of axillary lymph node metastases for SD cases *vs* clinical cases: estimate of the main effect of detection by screening according to tumour size category as obtained from model #3

**Tumour size (mm)**	**Adjusted OR^a^**	**95% CI**
2–7	0.05	0.01–0.39
8–12	0.36	0.23–0.56
13–17	0.40	0.28–0.58
18–22	0.95	0.64–1.40
23–27	0.59	0.33–1.04
⩾28	0.61	0.35–1.07

SD=screen detected; CI=confidence interval.

a OR=odds ratio; based on model #3, OR was computed as exp(*β*_1_) for the 2–7 mm size category, and exp^(*β*_1_+*β_j_*)^ (where *j*=2, …, 5) for the subsequent categories.

## Appendix A1

The investigators and participating institutions in the SCREENREG Working Group (Principal investigator: E Buiatti) were as follows: E Buiatti, S Bartolacci, Tuscany Regional Health Agency, Firenze; D Balzi, A Barchielli, Epidemiology Unit, ASL 10, Firenze; L Bucchi, F Falcini, A Ravaioli, Romagna Cancer Registry, Forlì; N Segnan, A Ponti, G Ronco, Screening Evaluation Unit, CPO Piemonte, Torino; S Patriarca, S Rosso, R Zanetti, Piedmont Cancer Registry, CPO Piemonte, Torino; A Frigerio, First Screening Centre, Torino; M Federico, R Negri, E Artioli, Modena Cancer Registry, Modena; V De Lisi, Parma Cancer Registry, Parma; S Ferretti, Ferrara Cancer Registry, Ferrara; E Crocetti, D Giorgi, E Paci, Tuscany Cancer Registry/Epidemiology Unit, CSPO, Firenze; M Vettorazzi, Veneto Cancer Registry, Padova; R Tumino, Ragusa Cancer Registry, Ragusa; and AC Finarelli, Department of Health, Emilia-Romagna Region, Bologna, Italy.
